# Multi‐Criteria Analysis for Effective Rain Water Harvesting Site Identification in Konso Zone, Ethiopia

**DOI:** 10.1002/gch2.202400333

**Published:** 2025-03-07

**Authors:** Fitsum Tsehay Bereded, Yohannes Mehari Andiye, Tarun Kumar Lohani

**Affiliations:** ^1^ Faculty of Hydraulic and Water Resources Engineering Water Technology Institute Arba Minch University Arba Minch P.O.Box 21 Ethiopia

**Keywords:** GIS, MCA, model builder, RWH, WOP

## Abstract

The Konso area of southern Ethiopia has limited resources and is highly vulnerable to climate change. Traditional agriculture practices in this region are adversely affected by water scarcity. The purpose of this study is to determine the most effective site for rainwater harvesting (RWH) through multi‐criteria analysis combined with Geographic Information Sysytem. The decision‐making criteria used in this study included rainfall, land cover, curve number, topographic wetness index, slope, distance from agriculture, lineament density, geology, and road and city distance. These criteria are categorized into five suitability levels based on their significance for rainwater harvesting using an analytical and hierarchical process (AHP). The study also mapped the restricted area, which includes the built‐up area and water accounting for ≈6% of the total area. The area with very high suitability for RWH is ≈658 km2, representing 28.3% of the total area. The suitability model is validated by cross‐checking existing RWH ponds with the appropriate map. It is found that most of the existing RWH ponds are located within high to moderately suitable zones, accounting for 92.6% of the total area. This research highlights the effectiveness of integrating MCA with GIS in identifying suitable RWH sites, especially in arid, semi‐arid, and data‐scarce areas. The weighted overlay process (WOP), available data, and methods are utilized to achieve this goal.

## Graphical abstract

Rainwater harvesting (RWH) has become a hot issue in the present scenario due to intermittent climate change, scanty rainfall, and insufficient surface runoff. Among such, the Konso area in the extreme south of Ethiopia is considered to find effective sites for RWH through multi‐criteria analysis combined with Geographic Information System. Various factors have been undertaken for MCA to find suitable sites. The criteria class is categorized into five suitability levels based on their significance for rainwater harvesting using an analytical and hierarchical process (AHP).

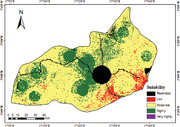

## 1 Introduction

The world water demand has radically increased due to massive population growth, global climate variation, urbanization, and industrialization of the countries. The arid and semi‐arid environments are the highest sufferers of water resource availability.^[^ ^1^ ^]^ These regions are characterized by irregular spatiotemporal distribution of rainfall. Proposing, implementing, and executing advanced technologies may enhance the better use of water resources management in most areas. RWH has shown renewed attention since 1980 as a rational solution to address water shortage problems in arid and semi‐arid regions. Ponds, pans, dams, terracing, percolation tanks, and Nala bunds are the most common types of RWH structures in arid and semi‐arid regions.^[^ ^2^ ^]^ However, executing the technologies is highly dependent on the site's suitability and their technical design.^[^ ^3^ ^]^

Different methodologies/tools have been used to select suitable sites for RWH development. They are Geographic Information System (GIS) with remote sensing (RS), hydrological model (HM) with GIS/RS, multi‐criteria analysis (MCA) integrated with HM and GIS/RS, and MCA integrated with GIS.^[^ ^4^ ^]^ Selecting the best method depends on various factors, like, hydrological processes, governing equations, assets, required data, and study area location. Among the methods, the development of MCA integrated with GIS is widely applied in arid and semi‐arid regions and data‐scarce areas.^[^ ^5^ ^]^

Site suitability evaluations for RWH not only depend on the methods/tools used but also the function of different input factors, such as topography, land use/land cover (LU/LC), rainfall, soil texture, soil depth, hydrology, hydro‐geology, socio‐economic, ecology, and environmental effects^[^ ^2^, ^4^, ^6^ ^]^ People living in arid and semi‐arid areas are exposed to inconsistent rainfall resulting to unpredictable droughts. As a result, the areas mainly suffered from water imbalance and poor hydrological and meteorological status.^[^ ^7^ ^]^

Lack of sufficient critical criteria and methodologies is one of the key challenges to properly allocating and plan RWH technologies in many parts of the world, particularly in arid and semi‐arid regions. The application of the MCA method has been more popular in the last few decades for site suitability studies. In the world, several studies have assessed the optimal site selection for effective RWH using MCA integrated with GIS. Among these, several studies,^[^ ^7^ ^]^ primarily focused on biophysical criteria, such as rainfall, slope, soil type, stream network, and land use land cover (LULC). Since 2000, most of the studies^[^ ^8^, ^9^, ^10^ ^]^ have attempted to combine socio‐economic criteria with the biophysical components as the main criteria for choosing RWH potential sites.

There are few studies carried out in Ethiopia^[^ ^11^, ^12^ ^]^ to locate suitable sites for RWH through MCA.

Many studies around the world have examined the best locations for effective rainwater harvesting (RWH) using MCA combined with GIS. Despite this, most of these studies have overlooked socioeconomic factors when selecting criteria. The majority of existing research focuses on a limited set of criteria, generally fewer than six. This narrow approach has often resulted in the exclusion of critical factors, such as the topographic wetness index and aquifer water productivity. Ignoring these important criteria can lead to significant issues during the planning phase of RWH systems. In this research, eleven criteria have been identified for analysis. This broader selection of criteria indicates that the present study has the potential to address the gaps left by previous research in terms of criteria selection. By including a wider range of factors, this approach aims to enhance the effectiveness of RWH planning and implementation.

This study focuses on the integration of a GIS with MCA to assess the suitability of locations for rainwater harvesting (RWH). The analysis considers various biophysical and socio‐economic factors, including hydro‐geological conditions and land moisture levels. Initially, a comprehensive MCA was conducted to evaluate different factors that influence the selection of ideal rainwater harvesting sites. This process involved collecting and analyzing data relevant to the specified criteria. Using GIS tools, the research produced maps that illustrate the suitability of different areas for rainwater harvesting based on these factors. The generated suitability maps were then compared against existing rainwater harvesting facilities to validate the findings. This validation process ensures that the identified suitable areas are practical and applicable for real‐world usage.

The study identifies suitable land areas for surface rainwater harvesting, featuring eleven key parameters that contributed to the suitability assessment. The main aim of this research is to enhance knowledge about GIS‐based multi‐criteria decision analysis and its application for effective rainwater harvesting in the Konso zone. The outcomes of this research may inform future water resource planning and implementation strategies. The suitability maps generated from this study are essential tools for hydrologists, decision‐makers, and planners. They will enable these professionals to quickly identify locations with the highest potential for rainwater harvesting, thereby facilitating better resource management in the region.

## 2 Experimental Section

### 2.1 Study Area

**Figure 1:**
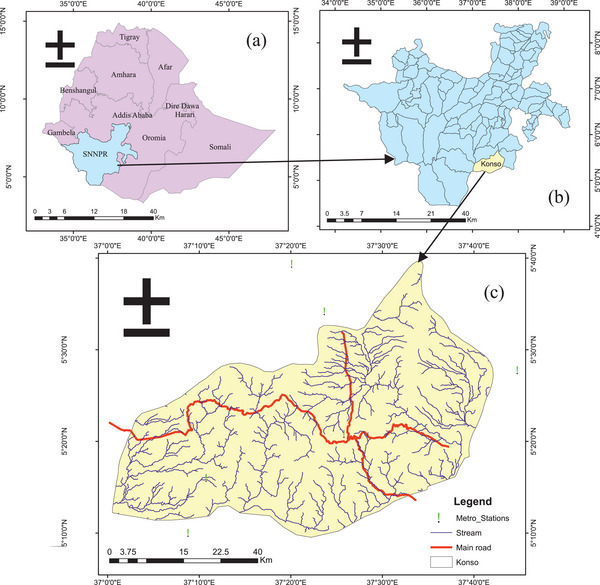
Location map of the study area; a) Ethiopian region, b) SNNPR, and c) Konso zone.

Konso is one of the zones found in the Southern Nation's Nationalities and People's Region (SNNPR) of Ethiopia situated in the Great Rift Valley, bordered by the Alle special woreda in the west, on the south the Oromia region, on the north the Dirashe special woreda, on the east Burji special woreda, and on the northeast Amaro special woreda (**Figure** **1**).

The Zone with a total area of 23 22km^2^ is located ≈600 km from Addis Ababa and 336 km from the SNNPR, geographically situated at 37° 29′ 0″ East Longitude and 5° 15′ 0″ North Latitude.

### 2.2 Topography and Slope

The topography is characterized by rugged surface features of hills and depressions, elevation ranging from 547 to 2094 m.a.s.l. Moreover, the slope ranges from flat to very steep from southwest to northeast.

### 2.3 Climate

Rainfall distribution follows a bimodal pattern with two rainy seasons per year. The first rainy season extends from March to May, whereas the second is between the end of August and November. The remaining months are dry with intermittent rainfall. The minimum monthly rainfall is recorded in January (24.2 mm). The average total annual rainfall of the area is between 400 mm and 1000 mm. The mean monthly temperature is 22.8 °C varying between the average of 17.4 °C (minimum) and 28.2 °C (maximum). The average monthly relative humidity of the area is estimated to be 60%. However, despite its pattern, the annual rainfall of the zone is widely known for its high variability and poor condition.

#### 2.3.1 Agro‐Climatic Zones

Agro‐climatic zones of the area were classified based on the traditional classification system according to the Ethiopian context. The Konso zone holds two major agro‐climatic zones, namely, Kolla and Woina Dega.

### 2.4 Soil Type and Geology

There are different types of soils as per their formations formation varying from place to place. The lowland of the area is dominated by Eutric Leptisols and Eutric Combisols soils, while the highland is covered by Eutric Vertisols and Humic Nitosols soils. The western side of the area is made up of predominantly metamorphic rocks such as gneiss and gabbros and local patches of syntectonic gabbros. Shallow deposits consisting of alluvium occur widely in the flat plain areas.

### 2.5 LULC Types

As per the LULC map of the study area, the mainland is dominated by agricultural land, water bodies, shrublands, urban areas, and barren land. The percentage of area coverage by each LULC type shows that 41.84% is covered by shrubland, 29.31% by agricultural land, 21.03% by barren land, and less than 8% is covered by water and urban areas.

### 2.6 Demographic and Socioeconomic Situation

Konso zone has a total population of 235 087 as per the 2007 census and there is no latest official record available for the population. Among these, 113 412 are male and the remaining 121 675 are female. The population density is 103.4 people per km^2^.

### 2.7 Data Collection

#### 2.7.1 Rainfall Data

To perform the objective of the study, rainfall data were collected from the National Meteorological Agency (NMA) of Ethiopia. A total of seven meteorological stations within or close to the study area were selected.

#### 2.7.2 Landsat Data

For this study, only OLI/TIRS C1 level‐1 landsat8 2019 atmospherically corrected Landsat image was selected for land cover mapping. The selection of the images was based on the criteria that the images have no cloud cover, i.e., less than 10% coverage.^[^ ^13^ ^]^ Landsat 8 sensor, Acquisition date 2019/03/13, spatial resolution 30 m × 30 m, and Path/Row 169/056 Landsat images (OLI) characteristics were used.

#### 2.7.3 Topographic Data

ASTER 30m × 30 m resolution DEM was downloaded from http://earthexplore.usgs.gov and Konso zone DEM was then extracted from this downloaded DEM data using ArcGIS 10.3. It was also projected into a coordinate system of WGS 1984_UTM _Zone_37°N. The DEM was used to delineate RWH parameters, such as, slope, lineament density, and topographic wetness index (TWI).

#### 2.7.4 Soil Data

Data related to soil type for the study area was obtained from the Ministry of Water Resource (MoWR). Soil type for Konso zone was then clipped using a spatial analyst tool (extract) of ArcGIS 10.3.

#### 2.7.5 Hydro‐Geological Data

Hydro‐geological data of Ethiopia was obtained from secondary data through related institutions. In this study, the data were obtained from Ethiopian Geological Survey. Hydro‐geology for Konso zone was then clipped using an analyst tool of ArcGIS 10.3. This data includes different hydro‐geological parameters, such as geology, lithology, and aquifer water productivity.

#### 2.7.6 Satellite Rainfall Data

The CHIRP product has the potential to generate a near‐real‐time satellite estimate at relatively high spatiotemporal resolution covering regions between 50°S and 50°N latitudes and all longitudes.

Based on the literature, the CHIRP rainfall product at daily and 0.05° * 0.05° spatial resolution for the year 1995 to 2019 was used (http://chg.geo.uscb.edu/data).

### 2.8 Data Analysis

#### 2.8.1 Checking Homogeneity of Stations

Peterson et al.^[^ ^14^ ^]^ recommended a method to apply homogeneity from neighboring stations that are supposedly homogeneous. The non‐dimensional of the months’ values are carried out as per (Equation 1).

(1) $$P_{i}=\frac{\bar{P_{i}}}{\bar{P}}*100$$

#### 2.8.2 Satellite Rainfall Biased Correction

Rain gauge data is mainly recognized as the most reliable source of information on rainfall but the high amount of missing data, combined with small coverage of the sampling area and inadequate spatial‐temporal rain gauge resolution, initiates to search for other data from the grid satellite rainfall time series. The long term daily precipitation for the period of 25 years (1995–2019) provided by the Climate Hazards Group Infrared Precipitation (CHIRP) data was chosen for its highest spatial resolution of 0.05° (≈5.3 km) and daily resolution (http://chg.geo.uscb.edu/data). Python (Spyder 3.7) software was used to convert the downloaded data into a time series (excel) format. A total of 108 grids were obtained and the bias of the CHIRP data of each grid was corrected using two steps; the rain gauge point scale data at seven stations for a period (1997–2007) was compared with CHIRP data at grid‐scale and correction factor a and *b* of the power transform method was obtained in a monthly basis. This is followed by the reliable monthly bias correction factors converted from station to grid data with the inverse distance weight approach^[^ ^15^ ^]^ (Equation 2).

(2) $$F\left(x\right)=\frac{\sum_{i=1}^{n}W_{i}\left(x\right)F_{i}}{\sum_{i=1}^{n}W_{i}}$$

where; $W_{i}\left(x\right)=\frac{1}{d{\left(X,X_{i}\right)}^{2}}$, X is an interpolation grid point, X_i_ is the known grid point (gauge location), d represents the distance between the known point X_i_ and the unknown point X (Centroid of grid elements), F(x) is the estimated value at X, *F_i_* is the available value at X and, n is the total number of known points.

Finally, correction was applied to all years (1995‐2019) by means of CHIRP data of the entire grid, using the monthly interpolated bias correction factor. The bias correction was estimated using the power transformation method^[^ ^15^ ^]^ (Equation 3).

(3) $$P=a*{\left(P_{\mathrm{CHIRP}}\right)}^{b}$$

where; P is daily CHIRP corrected rainfall amount, P_CHIRP_ daily CHIRP rainfall amount, *a*‐ pre‐factor such that the ratio of the mean of gauge observations to the mean of the transformed CHIRP, and b‐ factor calculated iteratively such that CV (Coefficient of variation) of gauge based observation ≥ CV of CHIRP.

After applying the bias correction to raw CHIRP data, the bias‐corrected CHIRP datasets were compared with observed rainfall data through performance measures, such as Regression coefficient (R^2^), Percent bias (P_bias_), and Nash Sutcliff Efficiency (NSE).

The regression coefficient (*R* ^2^) measures the degree of collinearity between corrected CHIRP and observed rainfall (Equation 4). R^2^ ranges in between 0 and 1. Higher values R^2^ indicates less error variance, and Values greater than 0.5 are usually appropriate.^[^ ^16^ ^]^

(4) $$R^{2}={\left[\frac{\sum_{i=1}^{n}\left(Poi-\bar{Po}\right)\left(Pci-\bar{Pc}\right)}{{\left(\sum_{i=1}^{n}{\left(Poi-\bar{Po}\right)}^{2}\right)}^{0.5}{\left(\sum_{i=1}^{n}{\left(Pci-\bar{Pc}\right)}^{2}\right)}^{0.5}}\right]}^{2}$$

The Nash Sutcliffe efficiency (NSE) is a normalized statistic that determines the relative magnitude of the residual variance compared to the observed data variance.^[^ ^17^ ^]^ NSE shows how well the corrected satellite data matches the observed data and with an interval between negative infinity and one (Equation 5). The latter revealed an accurate agreement between observed and corrected values.

(5) $$\mathrm{NSE}=1-\frac{\sum_{i=1}^{n}{\left(Poi-Pci\right)}^{2}}{\sum_{i=1}^{n}{\left(Poi-\bar{Po}\right)}^{2}}$$

The percentage of bias (P_bias_) measures the average tendency of the simulated data to be larger or smaller than their observed counterparts. The optimal value of P_bias_ is 0, with low magnitude values shows an accurate simulation. Positive and negative values of P_BIAS_ revealed that underestimation and overestimation bias, respectively.^[^ ^18^ ^]^

The P_bias_ is mathematically presented in (Equation 6).

(6) $$P_{\mathrm{bias}}=\frac{\sum_{i=1}^{n}\left(Poi-Pci\right)}{\sum_{i=1}^{n}Poi}$$

where; Po is observed or gauge rainfall (mm), Pc is corrected CHIRP data (mm), $\bar{Po}$ is observed mean rainfall (mm), $\bar{Pc}$ is corrected mean CHIRP (mm), n is the number of compared values for all equations.

### 2.9 Methodology of RWH Site Suitability Mapping

RWH's goals and related technologies are highly location specific and depend on physical, cultural, technological, and socio‐economic conditions. Thus, appropriate technologies are developed for specific place and cannot simply be replicated in other fields.^[^ ^19^ ^]^

Site suitability analysis for the development of RWH technologies is a multi‐criteria problem. Most of the existing RWH systems in the study area preliminary focused on surface runoff collection from open areas and farmers’ indigenous knowledge. Thus, the goal of this work was to generate a map for effective RWH zones by considering a multi‐input parameter excluded farmers’ indigenous knowledge. To perform this, different data including ground control points (GCP) and its methods were applied.

#### 2.9.1 Selection and Preparation of Appropriate Criteria

Several criteria were selected for the identification of potential zones for RWH (rainfall, LULC, soil texture, slope, distance from agriculture area, lineament density, curve number, Hydro‐geology, topographic wetness index, distance from city and distance from the main road). Biophysical and socio‐economic criteria were selected from those different studies which applied MCA integrated with GIS. Except soil texture and hydrogeology, all the input criteria were obtained and generated through step by step process and detail analysis was integrated with the appropriate software/tools.

##### Rainfall

Rainfall is the key parameter for any RWH system. Higher rainfall in any given region indicates higher possibilities for harvesting part of it.^[^ ^20^ ^]^ Moreover, rainfall maps with gridded data are usually used as an important input for many hydrological models and water resources management tasks.^[^ ^21^ ^]^ Thus, Because of the very high variability in distribution and amount of rainfall in the study area, it is very important to consider the rainfall as criteria for the identification of a potential zone. In this study, the kriging based interpolation method, i.e., ordinary kriging (OK) was implemented to generate gridded/raster rainfall maps from point rainfalls. Historical daily rainfalls of 108 point data for a period of 1995 to 2019 are obtained from corrected CHIRP (from section 3.4.2) and convert it to annual data, which are then used for the analysis. In general, the preparation of a rainfall map in grid format has two steps:

1) Select a more appropriate variogram model. The strength of kriging depends heavily on the proper choice of variogram model that shows the degree of spatial autocorrelation in the data set.^[^ ^22^ ^]^ Standard variogram models are generally used in OK. In this study, the three most commonly used standard variogram models in hydrology, namely, spherical, exponential, and Gaussian models were considered. The variogram model fitted with the lowest root mean square error (RMSE) and the highest correlation coefficient (R^2^) is selected as the best model.^[^ ^23^ ^]^ The variogram was calculated as:

(7) $$\Upsilon \left(d\right)\frac{1}{2N\left(d\right)}\sum_{i=1}^{N\left(d\right)}{\left(Z\left(X_{i}+d\right)-Z\left(X_{i}\right)\right)}^{2}$$

where; ϒ(d) denotes experimental variogram; Z(X_i_) and Z(X_i_ + d) are the rainfall values at corresponding sampling locations X_i_ and X_i_ + d, respectively, for a distance d and N(d) denotes the total number of data pairs.

2) After selecting best variogram model, ordinary kriging was implemented using GIS software for rainfall interpolation to generate gridded rainfall maps.^[^ ^23^ ^]^ In general, OK was computed as follows:

(8) $$Z_{OK}\left(X_{o}\right)=\left\{\begin{array}{c} \sum_{i=1}^{N}\omega_{i}^{OK}Z\left(X_{i}\right)\\ \sum_{i=1}^{N}\omega_{i}^{OK}=1 \end{array}\right.$$

where *Z_OK_* (X_o_) represent the estimated variable (rainfall in this study) at location X_o_; *Z*(X_i_) is observed value at location X_i_; ω_*i*_^*OK*^ represents the kriging weights linked with the sampled location X_i_ with respect to X_o_; N represents the total number of sample.

The kriging weights ω_*i*_^*OK*^ are obtained by solving an optimization scheme containing (N+1) simultaneous linear equations as given by;^[^ ^23^ ^]^

(9) $$\sum_{i=1}^{N}\Upsilon_{std}\left(d_{ij}\right)\omega_{i}^{OK}+\mu^{\mathrm{OK}}=\Upsilon_{std}\left(d_{i0}\right)\;\mathrm{for}\;j=1\;\mathrm{to}\;N$$

(10) $$\sum_{i=1}^{N}\omega_{i}^{OK}=1$$

where: ϒ_*std*_(*d_ij_*) and ϒ_*std*_(*d*_*i*0_) represent the variogram values that come from the standard variogram models for the distance d_ij_ and d_i0_, respectively; d_ij_ denotes the distance between sampling point's X_i_ and X_j_; d_i0_ denotes the distance between the sampling point X_i_ and the target location and µ_ѳ_^OK^ represent the Lagrange multiplier.

##### LULC Map

In preparation for the LU/LC map, RS image classification is generally the main target. Image classification involves several steps, for instance, collecting training samples, selection of appropriate images, preprocessing, selection of suitable classification algorithm, and accuracy assessment.^[^ ^24^ ^]^ In this study, the LU/LC type was prepared by using a Google earth engine (GEE) online java programming application. The landsat image used in this study was atmospherically corrected. Thus, no need to landsat image preprocessing.

##### Field Work

Several field visits, aided using GPS, were carried out to collect GCPs. The data collected during the fieldwork were used for two key purposes: (1) to pick types of the major LU/LC in the study area, which support to prepare LU/LC classes, (2) to associate the ground truth of a specific type of LU/LC with its image characteristics, which helped to classify images and accuracy assessment. However, at a time of fieldwork, reaching all areas to collect GCPs is impossible because the study area is very difficult with a very steep slope and very challenging to access, in particular the majority of areas which are very far from the main road. Moreover, during fieldwork, the peace and stability of the area was bad, i.e., there were conflicts between different tribes. Thus, in that case, Google earth software was used to collect GCP data. From the total number of the GCP data, 80% of data points were used for training of image classification algorithm, and 20% for accuracy assessment.

##### Identifying LU/LC Classes

Several LU/LC types were identified during the field visit and Google earth. Later the classes were reduced into five LU/LC classes by merging the same types of classes with similar characteristics. The most important requirement for the classes in the study area is homogeneity within a certain location.

##### Image Classification

LU/LC mapping through satellite images usually requires a certain classification algorithm. Different kinds of classification algorithms are available based on the method of classification. The classification and regression trees (CART) algorithm was applied for this study.

##### Accuracy Assessment

The accuracy of LU/LC classifications can be expressed quantitatively by building and interpreting a classification confusion error matrix. In this study, a confusion matrix together with descriptive statistics namely, user‘s accuracy, producer‘s accuracy, overall accuracy, and kappa coefficient were produced which helped to understand the accuracy of the classification. Accuracy assessment was conducted between the classified data and the reference data. The overall accuracy was calculated by dividing the correctly classified pixels by the total number of ground truth pixels. Another quantitative measurement of the accuracy of LU/LC classification is the Kappa coefficient.

Generally, the Kappa coefficient was computed as:

(11) $$K=\frac{P_{o}-P_{c}}{1-P_{c}}$$

where; P_o_ the proportion of observed agreements and P_c_ proportion of agreement expected by chance.

##### Slope

In this study, a DEM with a 30‐m resolution was properly used to generate a slope map. The resulting slope map was then divided into five slope classes. After reclassification, a possible rate was typically set for each slope class based on their relative importance for effective RWH technologies.

##### Distance from Agriculture Area

RWH has agricultural uses. It can be used for watering garden in homes and crop plants in agricultural fields. Therefore, one of the criteria for identifying potential areas for RWH was distance from agricultural lands. In this study, the distance from agriculture area was derived from the LU/LC map using ArcGIS tool.

##### Soil Texture

According to the Ministry of Water Resources of Ethiopia (MoWR) soil database, four soil texture classes are noted in the study area; namely, loam, sandy loam, loamy sand, and clay. The higher percentage of the soil was found to fall in clay soil, i.e., 50.8% of the total area, which translated to very low infiltration and high runoff leading to a high curve number. As a result, the soils within the study area were found to be useful for surface water harvesting and the generation of runoff. The rest 26% of the area comprise Loam, 20.3% is loamy sand and 2.6% is sandy loam. In terms of location, clay soil dominates both the central and southern parts of the area, while both loam and loamy sand dominate the eastern part of the area.

##### SCS Curve Number

The higher the curve number, the higher the excess rainfall on the area and vice versa.^[^ ^25^ ^]^

The curve number distribution map was generated based on soil data from MoWR, and the prepared LULC map of the study area. The soil texture was extracted from the MoWR soil database. As a result, four classes of soil texture were obtained, namely loam, sandy loam, loamy sand, and clay soil. Following this, the hydrological soil group (HSG) was then derived from the soil texture as shown in **Table** **1**.

**Table 1:** Hydrological soil group of Konso zone.

No	Texture class	HSG
1	Loam	B
2	Sandy loam	A
3	Clay	D
4	Loamy sand	A

The curve number was then calculated in ArcGIS 10.3 through the union process of the LULC and HSG (**Table** **2**).

**Table 2:** CN Lookup table adapted from (SCS‐TR55, 1986).

No	LULC	HSG			
		A	B	C	D
1	Agricultural land	67	77	83	87
2	Shrub land	48	62	73	78
3	Urban	77	86	91	94
4	Water	100	100	100	100
5	Barren land	68	79	86	89

##### Hydrogeology

Recently, knowing groundwater productivity zones plays an increasingly significant role in the sustainable management of surface water resources of all over the world. Thus, in this study, groundwater productivity of aquifer was considered as one of the criteria for RWH site suitability analysis.

According to the Ethiopian geological survey database, five major geological classes were noted in the study area, namely, Quaternary (Qa), Pleistocene (Ql), Pliocene (Tg), sandstone (Tt), and Canyon (hm). The most dominant geological formation is a canyon, which covered about 63.2% of the study area. About 15.3% of the area is local sandstone found in the southern part of the area. The other three geological categories are; Pleistocene (1.3%), Pliocene (19.4%), and Quaternary (0.6%). Moreover, The Ethiopian geological survey Hydro‐geological map indicates

##### Lineament Density

Lineaments would be highly important when groundwater recharge is to be conducted and least suitable for surface RWH because they would encourage leakage or infiltration. In general, the higher lineament density has greater permeability and vice versa. In this study higher scale was set to an area that has low lineament density and a small scale was given where an area has higher lineament density.

Liu et al.^[^ ^26^ ^]^ stated that DEM is useful in lineament studies; DEM was therefore used for lineament identification and extraction in this research. Hill Shad relief images from DEM were created using the GIS tool. Lineaments were identifiable in shaded relief images using differences in sun illumination. The lineaments were then digitized in a GIS software and the lineament density was generated using the line density tool in arc GIS.

##### Topographic Wetness Index

In this paper, to represent the spatial variability of water flow and water stagnating across the study area, the steady‐state topographic wetness index was used. Topographic wetness index is purely based on topography and it is a function of the upstream contributing area and slope.^[^ ^27^, ^28^ ^]^ The TWI was generated in ArcGIS 10.3 software. Using DEM 30 m resolution, the flow accumulation and slope (in radian) were prepared separately through surface analysis and hydrology tools. Thus, TWI was calculated in GIS as:

(12) $$\mathrm{TWI}=\ln\left(\frac{\left(a+1\right)*d}{Tan\left(\beta \right)+C}\right)$$

where: “d” is DEM cell size (30m), ɑ is flow accumulation, β is slope in radian and C is constant value 0.001.

##### Distance from City

The RWH should be situated far away from public utility areas. For instance, from the residential area, commercial, institution, industry, hospital, and so on. Because wastes from those areas may be discharged into harvested water, they are likely to pollute the stored water. Understanding the concept of distance from the city is critically important to minimize these socio‐economic impacts. In this study, distance from the city was considered as criteria. 5 km radius was assumed a restricted area and proposed any RWH technologies within this section is not allowed.

##### Distance From Road

According to Mahmood and Al‐Ardeeni,^[^ ^29^ ^]^ the minimum recommended distance to roads is 100 m. In this study, distance from the road was considered as criteria. It was achieved by first extracted a road map from Google earth and digitized in GIS. The only main road was taken and the distance from the road was then calculated using spatial analysis tool in raster format.

#### 2.9.2 Suitability Level for Each Criterion Class

Each class of criteria map was reclassified into five comparable units, i.e., suitability classes such as; 1 (very low suitability), 2 (low suitability), 3 (moderate suitability), 4 (high suitability), and 5 (very high suitability). The suitability classes are then used as a base to generate the criteria annex‐A shows the assigned scores of each class based on discussions and interviewed with experts and the published information.

#### 2.9.3 Establish Weight of Criteria

All the criteria are not equally important for the proper identification of the potential zone of RWH structure. Thus, relative weight was computed to each criterion. To compute the weights, (**Table** **3**) the analytical hierarchy process (AHP) used to create a pair‐wise comparison matrix [A].^[^ ^30^ ^]^

**Table 3:** Pair wise comparison matrix.

	RF	DA	TWI	LULC	Geo	ST	SL	CN	LD	DC	DR
RF	1	1	2	3	3	5	5	5	7	9	9
DA	1	1	2	2	2	4	5	4	6	7	7
TWI	1/2	1/2	1	2	2	3	5	4	5	6	6
LULC	1/3	1/2	1/2	1	3	3	4	3	3	4	5
Geo	1/3	1/2	1/2	1/3	1	2	3	2	2	3	5
ST	1/5	1/2	1/3	1/3	1/2	1	2	2	2	3	4
SL	1/5	1/5	1/5	1/4	1/3	1/2	1	1	2	2	3
CN	1/5	1/2	1/4	1/3	1/2	1/2	1	1	2	2	3
LD	1/7	1/6	1/5	1/3	1/2	1/2	1/2	1/2	1	2	2
DC	1/9	1/7	1/6	1/4	1/3	1/3	1/2	1/2	1/2	1	1
DR	1/9	1/7	1/6	1/5	1/5	1/2	1/3	1/3	1/2	1	1

Abbreviations: RF, Rainfall; CN, Curve Number; TWI, Topographic Witness Index; LULC, Land Use Land Cover; ST, Soil Texture; Geo, Geology; DA, Distance from agriculture area; LD, Lineament Density; SL, Slope; DR, distance from road; DC, Distance from city

Once the pair‐wise comparison matrix was established, the normalized pair‐wise comparison matrix A_norm_ was extracted from [A] by making the sum of the entries on each column of [A] equal to one.^[^ ^31^ ^]^ The value of A_norm_ was calculated as:

(13) $${\bar{A}}_{jk}=\frac{A_{jk}}{\sum_{l=1}^{m}A_{lk}}$$

At last, the weight (W) of each criterion (that is an m‐dimensional column Eigen vector) was computed by averaging the entries on each row of A_norm_. Mathematically W is represented as;

(14) $$W_{j}=\frac{\sum_{l=1}^{m}\hat{A}jl}{m}$$

where: “m” is the total number of criteria (11 in our case), j and k prefix, ${\bar{A}}_{jk}$ is normalized pair wise comparison matrix and, “W” is weight of criteria's.^[^ ^31^ ^]^

#### 2.9.4 Checking the Consistency of Pair‐wise Comparison Matrix

Consistency Index (CI) is the quantitative measure to judge whether the comparison matrix is consistent or not. It is obtained by first calculating the scalar *λ*_max_ as the average of the elements of the vector whose j^th^ element is the ratio of the j^th^ element of the vector A*W to the corresponding element of the vector W.^[^ ^32^ ^]^ Mathematically CI is read as:

(15) $$\mathrm{CI}=\frac{\lambda_{\max}-m}{m-1}$$

Note: a decision‐maker should always obtain CI = 0 for perfect consistency. However, small values of inconsistency may be allowed when CR<0.1.

(16) $$CR=\frac{\mathrm{CI}}{RI}$$

where: CR is consistency ratio; RI is Random Index which is the consistency index of a randomly generated pair‐wise comparison matrix. In evidence, the RI is affected by the number of criteria being compared.^[^ ^32^ ^]^

#### 2.9.5 GIS analysis and Generation of Suitability Map

The model builder was developed in ArcGIS 10.3 as shown in Annex‐D. For each place, the cells in each input theme or layer at that place are weighted and then the multiple layers were also overlaid to generate the output image.^[^ ^33^ ^]^ The suitability level (S) for cell i mathematically read as:

(17) $$\begin{array}{ccc} & & S_{i}=\left(W_{1}*S_{1i}\right)+\left(W_{2}*S_{2i}\right)+\\ & & \cdots \cdots +\left(W_{n}*S_{ni}\right)=\sum_{j=1}^{n}W_{j}S_{ji} \end{array}$$

where: S_i_ is suitability at cell i; *W_j_* is relative importance weight of criteria (“j” starts from 1 to 11 in this study); *S_ji_* suitability level of cell i in the criteria “j”.

Therefore, the higher the suitability values *S_i_* of a given cell, the more appropriate it is for RWH structures.

### 2.10 Validation

Validation revealed that whether the method and data used to perform the suitability map is reliable or not. This was achieved by cross‐checking the suitability map with the location of existing RWH ponds. The proper locations of existing RWH ponds were carefully read through the global positioning system (GPS) instrument and then incorporated in the ArcGIS 10.3 software for validation analysis.

## 3 Results and Discussion

### 3.1 Validation of Corrected CHIRP Dataset

**Figure 2:**
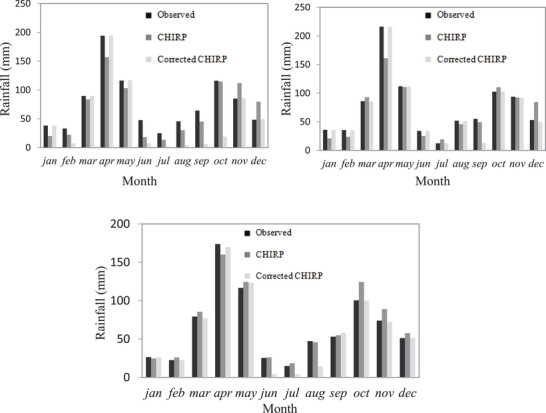
Station to grid comparison of the monthly average rainfall of observed, uncorrected, and corrected for the period 1997–2007 a) Arfayde, b) Kolme, and c) Konso.

As aforementioned, the rainfall data used in this study was the historical data obtained from gauge measurements, and satellite‐based rainfall estimation products; CHIRP for a period of 25 years from 1995 to 2019. Further, to evaluate the monthly variation between the two datasets, the mean monthly rainfall patterns of the CHIRP satellite and the rain gauge stations for Konso, Kolme, and Arfayde stations from 1997 to 2007 are presented in **Figure** **2**. The highest negative difference between the two datasets was obtained in April, with values of −37.1, −55.3, and −13.7 mm for the Arfayde, Kolme, and Konso stations, respectively. This implies that satellite‐based rainfall data underestimated the rainfall data in this month. The conclusion is that the degree of the CHIRP satellite rainfall data bias varied at a monthly scale across the study area. Thus, monthly bias correction for satellite data was critically important to minimize their differences.

**Figure 3:**
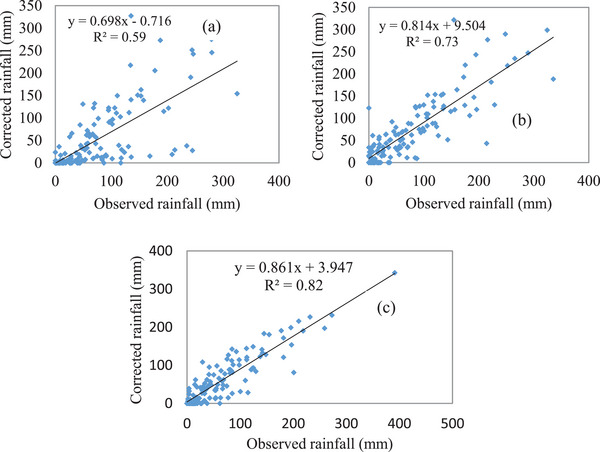
The scatter plot for the monthly rainfall comparison between corrected and observed at period 1997 to 2007 a) lower zone (Arfayde), b) medium zone (Kolme), and c) upper zone (Konso).

The scatter plot of observed rainfall at monthly time scales for three meteorological stations, namely, Arfayde, Kolme, and Konso with the overlying corrected CHIRP rainfall data for the years 1997 to 2007 (**Figure** **3**). The results indicate that there is a very good correlation between corrected CHIRP data and observed rainfall for Kolme and Konso stations by 0.73 and 0.82, respectively.

Further, the performance of the corrected CHIRP product was evaluated by comparing it with three rain gauge (**Table** **4**).

**Table 4:** Result of performance measurement for three stations.

Performance measure	Lower zone (Arfayde)	Medium zone (Kolme)	Upper zone (Konso)
P_BIAS_	0.11	0.06	0.08
NSE	0.8	0.94	0.93
R^2^	0.59	0.73	0.82
ME	23.4	0.078	5.12
RMSE	37.89	22.56	12.06

### 3.2 Generated Maps of Input Criteria

#### 3.2.1 Rainfall Map

##### Variogram Model for Spatial Auto‐Correlation

In this study, to fit the experimental variogram, three key standard variogram models namely, Gaussian, exponential, and spherical models were selected for all the available data sets. The variogram parameters that result in the best fitting performance based on RMSE and R^2^ measures were picked as the best‐fitted model. Moreover, the semi‐variogram parameters and fitting performance of the standard variogram models for annual rainfall data are shown in **Table** **5**. The results show that for annual rainfall, the spherical variogram model has the lower RMSE (77.66) and largest R^2^ (0.93) values compared to the exponential and Gaussian variogram model as shown in Table 5.

**Table 5:** Performance measurement and variogram parameters of ordinary kriging.

Variogram Model	Performance Measure						
	ME	RMSE	RMSSE	MSE	R^2^	Range [km]	Sill
Exponential	0.24	78.77	0.49	0.0024	0.929	0.67	1.18
Gaussian	−0.64	80.19	0.72	0.0003	0.926	1.129	0.32
Spherical	−0.59	77.66	0.51	−0.0018	0.93	2.435	0.76

**Figure 4:**
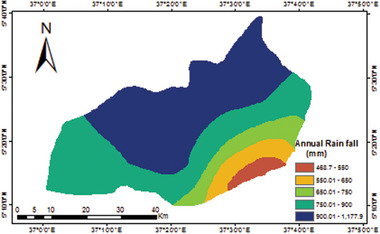
Mean annual rainfall depth map of Konso zone (1995–2019).

The 25 year (1995 to 2019) average annual rainfall distribution map of the Konso zone is shown in **Figure** **4**. The map was developed by using ordinary kriging through the standard spherical variogram model. The map revealed that there is a significant variation in patterns of rainfall across various sections of the study area. The mean annual rainfall varies across the study area from about 469 mm in the lower zone segment to 1178 mm in the upper Konso zone segments. In terms of direction, the southern part of the area is highly exposed to a dry condition in general, northern parts of the area have high rainfall value, while both western and eastern parts of the area have relatively medium annual rainfall patterns. Therefore, the most northern section of the study area is more appropriate for RWH development in terms of rainfall.

#### 3.2.2 Classified LU/LC Map

Without a quantitative statement about their accuracy, the LU/LC maps are not very useful. This study was used only 20% of the collected GCP for the validation of the map which is generated in this study. The accuracy of the maps is presented as an error matrix as shown in **Table** **6**. The numbers along the diagonal of the matrix show that the number of reference pixels that are accurately classified by the algorithm. The values outside the diagonal indicate misclassifications. The classification confused only some of the LULC. For instance, four shrub pixels were classified as water due to the reflectance similarity of some agriculture with shrubs.

**Table 6:** Confusion matrix of 2019 LU/LC classification.

	Reference Data						
Classified Data	UL	AL	WB	SL	BL	Σ	User Accuracy [%]
UL	3	1	0	0	0	4	75
AL	0	12	3	0	0	15	80
WB	0	1	56	7	0	64	87.5
SL	1	0	4	179	4	188	95.21
BL	0	3	3	8	33	47	70.21
Σ	4	17	66	194	37	318	
Producer Accuracy [%]	75	70.6	84.8	92.26	89.2		

Abbreviations: UL, Urban land; AL, Agricultural land; WB, Water body; SL, Shrub land; BL, Barren land

The accuracy indicators revealed that the overall accuracy is 89% and the Kappa coefficient is 0.81. This implies that the classification accuracy indicators are within the acceptable range compared to the recommended individual accuracies of at least 70%; overall accuracy is more than 85% and Kappa coefficient is at least 75%. This value is better because of the higher spatial resolution of the reference data used.

Therefore, based on the matrix result, visual interpretation, and comparisons of the areal extent of the classes, Google earth engine (GEE) Java online programming software integrated with the Classification and Regression Trees algorithm is an effective method for the preparation of LU/LC map.

**Figure 5:**
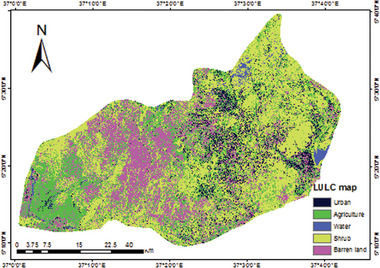
Land use land cover map of Konso zone in the year 2019.

The LU/LC map shows five (Urban, Agricultural land, Water, Shrub land, and Barren land) classes of LU/LC were generated. The land cover map in **Figure** **5** shows the land class coverage of ≈6.36% of the Konso zone was covered by urban or built‐up area, 29.31% by Agricultural land, 1.44% by water, 41.84% by shrub land and 21.03% by barren land. In terms of direction, agricultural land dominantly covers the western parts, shrubland is found in most parts of the study area, barren land is found in the western and southern, urban in the central part, and water is found in the eastern part of the study area. In general, currently, the study area is typically dominated by shrubland.

#### 3.2.3 Map of Curve Number

**Figure 6:**
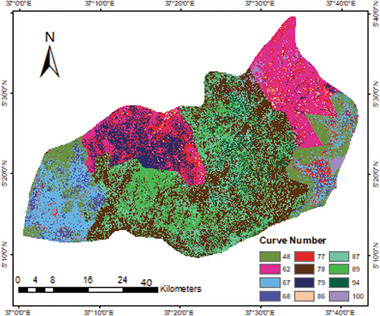
Generated curve number.

The curve number between 48 and 100 was obtained in the study area as shown in **Figure** **6**. The smallest curve number was estimated in the shrubland area, while the maximum CN value was obtained in the water body. The present study also revealed that the CN value of urban/built‐up areas varies from 77 to 94, depending on the quantity of impervious areas and the type of soil available. Moreover, the percentage of coverage by each CN value indicated that 33.5%, 35.8%, 25.5%, and 5% of the total area have CN within 48 to 68, 68 to 79, 79 to 89, and 89 to 100, respectively.

Overall, the mapping analysis shows most CN values in the Konso zone are high, i.e., 67% of the total area has a range of 68 to 100. Consequently, the available soils within the study area were proven to be effective for RWH and producing a lot of runoff.

#### 3.2.4 Map of Others Input Parameters

**Figure 7:**
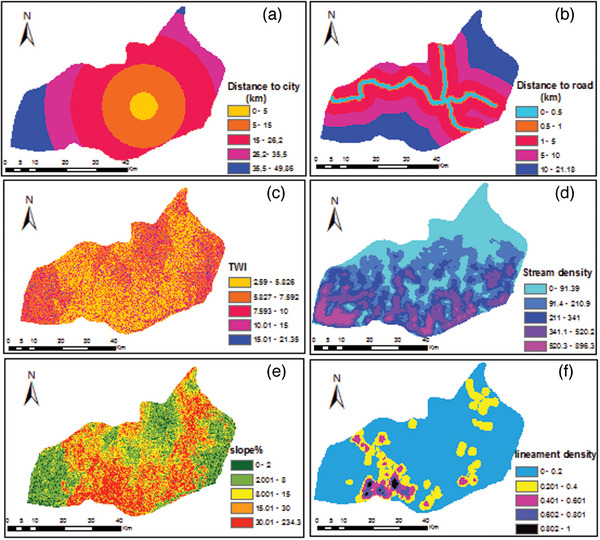
Map of other input criteria a) distance to city, b) distance to road, c) TWI, d) distance to agriculture area, e) slope, and f) lineament density.

The slope is another parameter for identifying an effective site for RWH development. The resulting slope map is presented in **Figure** **7**e. The result shows the highest area percentage of the study area falls within the flat to gently sloping category (2% to 8%) covering 33.2% of the total area, followed by moderately steep (15% to 30%) occupying an area of 22.8%. A small area was found on the generally flat slope category (0 to 2%) covering 4.8%. Usually, flat and gentle slope classification categories offer favorable to rain collection sites, because such flat areas allow water to accumulate and not flow away and, thus, help to concentrate the runoff of rainfall from a given storm, naturally. Moreover, the land which covers 18.2% is gently rolling and 21.1% is occupied by steep to very steep slopes. The southern part is characterized by steep slopes and both the eastern and western part is dominated by flat to gentle slopes.

The topographic wetness index (TWI) map was generated by the Arc map spatial analyst tool. As illustrated in Figure 7c, five TWI classes were identified. This generated map of TWI indicated that the spatial distribution of water stagnates across the study area. The higher TWI value is considered highly suitable for RWH and vice versa. The percentage area coverage shows that 91.2% of the study area was found within TWI value less than 10. Thus, the majority of the area has less water moisture capacity. The remaining 8.8% of the study area falls within 10 to 21.35 and is located in the eastern part of the area.

The stream density as illustrated in Figure 7d, has five categories which vary from 0 to 896.3. The lineament density information that was produced in this research is presented in Figure 7f. The result shows that denser lineament has occurred in the southern part of the study area, which implies a high degree of hydraulic interconnection between the lithological units. Moreover, moderate and low lineament density concentrates in the central and eastern parts, indicating a zone with low levels of groundwater recharge. Based on the literature the higher lineament density is not suitable for RWH. Lineaments are underlined by zones of focalized weather, as a result of increased permeability and porosity.^[^ ^34^ ^]^

Another factor for the assessment of land suitability for RWH in the Konso zone was the distance from the city. It was one of the minor key parameters for deciding the optimal site for RWH ponds. The resulting map is presented in Figure 7a. The map was generated by using Arc GIS 10.3 with the Euclidean distance tool. Accordingly, the obtained map was classified into five categories with different intervals. Each class shows the distance (km) from the center of the city to a point located inside the class. The first class (0 to 5 km) represents a more built‐up/urbanized area. Based on the FAO (2006) guideline, RWH sites should not be proposed within such kind of area. Thus, this class was considered a restricted area (buffer zone). The remaining four classes were taken for land suitability analysis in spatial multi‐criteria.

The last input parameter for site suitability analysis in this study was the distance from the main road (Figure 7b). The map revealed the Euclidean distance from any point in the study area to the center of the road. Rank was set for each class based on the requirement that the road should be far away from the RWH site. The reason behind this is to prevent risk on the road from an unexpected flood and avoid any conflict. In this study, 100 m from the left and right side of the road was taken as a buffer zone. Moreover, a greater distance between the road and RWH is not recommended because of accessibility. Therefore, a higher rank was given for the second class (0.5 to 1 km).

##### Weight of Criteria's

**Figure 8:**
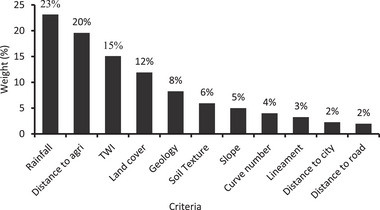
Importance weights of the criteria in AHP.

The suitability map for RWH was derived from the criteria maps and must be linked to the findings of the analytical hierarchy process, based on the comparative importance of each criterion that was evaluated. **Figure** **8** reveals the calculated Eigen values or the relative importance weight of each criterion in percentage. The result shows that the most important factor for RWH site identification is rainfall (23%). This is followed by a distance to the agriculture area (20%), TWI (15%), and LU/LC (12%). The remaining seven factors, such as soil texture, geology, slope, curve number, lineament density, distance to the city, and distance to the main road, share only 30% influence.

As evaluated from Equation (16), the consistency ratio (CR) of the pair‐wise comparison matrix is 0.03, which shows that the cumulative judgments obtained from the pair‐wise comparison matrix from Table 8 are adequate, i.e., the CR value is lower than or equal to 0.1. Thus, the degree of inconsistency is tolerated.

#### 3.2.5 Suitability Map of RWH

**Figure 9:**
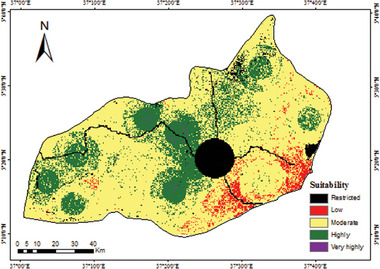
Site suitability map of RWH.

The RWH suitability map was generated in the model builder by combining various input key criteria maps through weigthed overlay process. As a result, four suitability classes were obtained, such as very highly suitable, highly suitable, moderately suitable, and low suitable. The spatial distribution of suitability classes (**Figure** **9**). There is no very low suitable zone in the study area, as evidence that there is no grid cell value in the resulting suitability map. Moreover, the restricted area was mapped based on FAO (2006), including the built‐up/urban area, and water body which take ≈6.0% of the total area. FAO (2006) guidelines suggest that RWH system should not be located within natural forests, protected areas, ecologically sensitive areas, and built‐up areas.

Furthermore, the percentage area coverage by each suitability class shows 0.1% and 28.1% of the entire area is very highly and highly suitable for RWH development, respectively, whereas 63.2% of the area is moderately suitable, and lastly, 2.6% is less suitable. In terms of direction, the most suitable categories are situated in western and northern parts, which have flat to gentle topography (slope less than 8%) and low elevation and more than 800 mm per year mean rainfall, which makes it adequate for rainfed agriculture. Both clay and loam soils are the major soil texture classes in the areas of very high and high suitability. According to the Ethiopian geological survey map, the aquifer groundwater productivity ranges from low to medium productivity in these suitability classes.

On the other hand, the low suitability zone fell under the eastern parts, which have a curve number of less than 68 and annual rainfall of less than 500 mm. The main hydro‐geological formation in this suitability class is the Quaternary formation, which has high groundwater productivity (**Table** **7**).

**Table 7:** Percentage coverage of each suitability class.

No	Suitability class	Area [km^2^]	Coverage [%]
0	Restricted area	107.8	6
1	Very low suitability	0	0
2	Low suitability	61.7	2.6
3	Moderate suitability	1481.1	63.2
4	Highly suitability	655.5	28.1
5	Very high suitability	2.4	0.1

##### Suitability Relative to LU/LC

The LULC type was one of the requirements for the selection of possible areas for RWH structures. However, all types of LU/LC are not suitable for RWH development. The RWH structure meant for crop production should be near agricultural lands. Based on this reason, the agricultural land was ranked higher than the other types of LU/LC. The classification of suitability by LULC type, is expressed as a percentage of each land cover type. This was achieved by overlaying the resulting suitability map with the prepared LU/LC map. The result indicated that the majority of very high to highly suitability classes fell under agricultural lands, shrub land, and barren land ≈32.83%, 32.3%, and 25.55% of the total areas, respectively. Moreover, according to FAO's (2006) recommendation, RWH structures should not be located within the water body and built‐up area. Thus, in this study, water and urban/built‐up areas have no land cover classified as very highly suitable to highly suitability.

On the other hand, a higher percentage of medium suitability levels fell under shrubland (45.6%), followed by agricultural land (28.7%) and barren land (20.2%). Therefore, the suitability map significantly improves the agricultural performance of the Konso zone farming activities, because 38.5% of very highly suitable classes are fall within agricultural areas.

##### Validation of Suitability Map

After the site suitability map is prepared, validating the resulting potential map is critically important to decide whether the database and methods used were good or not. Although it is believed that a detailed document about the existing RWH is needed to validate, most existing RWH ponds are made with the traditional knowledge of the community. As a result, deep information about existing RWH could not be. In this case, validation can be performed by simply collecting the locations and rating the performance of existing RWH ponds.^[^ ^12^, ^35^ ^]^ A total of 41 existing RWH pond locations were collected during fieldwork using GPS. For the validation, the surveyed existing RWH ponds were first rated into two classes; success and failure. As a result of the field observation, most of the existing RWH ponds have been proven to have minor problems and have been categorized as a successful class, because the community has strong indigenous knowledge of RWH. Therefore, the assumption made during the validation was that if these existing RWH systems, which were classified as success categories were to be located in the very high to medium suitability areas of the derived suitability map, the output of the suitability model developed could be used in future for RWH development.

The validation result indicates that most of the existing RWH systems were categorized as successful, i.e., (48.78%) fall within the highly suitable areas followed by moderately suitable (43.9%). Just 7.32% of existing structures were found within the restricted area. The fact that almost all of the existing RWH technologies fall within a high to medium suitable (92.68%) reveals that the dataset and methods used to build the suitability model can be accurately used to foretell optimal RWH structure sites. In other words, the designed suitability map strongly agrees (92.68%) with the locals indigenous knowledge.

Moreover, the number of existing RWH structures per woreda level with the corresponding suitability classes is shown in **Table** **8**. For example, out of the six existing RWH ponds that Kolme Woreda had read, all of them fell within the medium suitability class. From 20 existing RWH structures read by Kena Woreda, 11 fell in the high suitability class, and 9 fell within the medium suitability category. Two existing ponds had been located in a restricted area of the Karat city Administration and one in Karat Zuria.

**Table 8:** Comparison of suitability categories and existing RWH locations per woreda level.

Woreda name	Number of RWH	Very high	High	Moderate	Low	Very low	Restricted
Kolme	6	0	0	6	0	0	0
Kena	20	0	11	9	0	0	0
Karat zuria	13	0	9	3	0	0	1
Karat town	2	0	0	0	0	0	2
Total	41	0	20	18	0	0	3
Percentage of fall [%]		0%	48.78%	43.9%	0%	0%	7.32%

### 3.3 Summarized Site Selection

**Figure 10:**
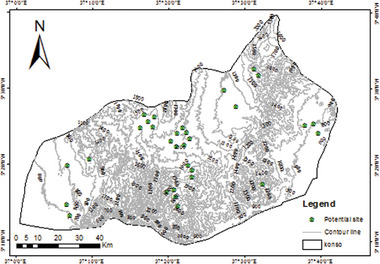
Potential RWH sites.

This study revealed that the generated RWH suitability map has a varied range of suitability, i.e., less suitable to very highly suitable. However, proposing and implementing the RWH pond on all suitability classes is uneconomical and impractical. Thus, limiting the number of sites is critically important. Only a very high suitability class (represented in the fifth class) is considered to impede possible sites in the study area. The result shows thirty‐one (31) potential sites were obtained. These sites have been digitized and shown in **Figure** **10**. The recommended type of RWH is storage ponds, which are possibly used for watering livestock, recharging of groundwater, irrigation, or some other purpose other than drinking water.

### 3.4 Comparison of Current Findings Against Past Similar Studies

The current study presents the utilization of MCA integrated with a GIS application to identify site suitability maps. In many cases, MCA were integrated with GIS for surface RWH optimal site identification.^[^ ^7^, ^8^, ^12^, ^35^, ^36^ ^]^ Thus, it is important to compare and examine the current findings with previous similar studies. The comparison was made in a different aspect. For instance, input criteria selection. In this study, an effective surface RWH site was identified by considering biophysical and socioeconomic factors. The factors were selected in consideration of free availability, moderate spatial resolution, and proven applicability in a wide range of arid and semi‐arid environments. Most of the input criteria used in this study were similar to past studies. Some additional criteria like TWI, and lineament density were included in the current studies to make a result more reliable. For all past studies, Scale was assigned to each criterion based on their relative importance for RWH. The resulting weight of each criterion indicated that rainfall was the highest important parameter for RWH site suitability in most studies. This is the same as the current study.

To examine the performance of the suitability model, test or validation of the suitability map was done in this study using information obtained from existing RWH structure locations. This procedure is the same as the previous studies.^[^ ^35^ ^]^ reported that the decision support system categorization is strongly agreed by 81.4% with the existing RWH. In the same way, the result of current study was 92.68%; this result is better than the previous similar studies because increasing input criteria made it strong. In general, the comparison shows there is a remarkable agreement between the past and the present study. Thus, it can be concluded that the MCA integrated with GIS method is the best way for site suitability investigation.

Previous studies indicated four suitability classes for land evaluation in relation to rainwater harvesting (RWH). These classes are categorized as highly suitable areas, suitable areas, moderately suitable areas, and less suitable areas.^[^ ^37^ ^]^ In the current study, a RWH suitability map was produced. This map was validated through two methods: ground verification using Google Earth Pro and an actual field trip to the study site. Validation is essential to ensure the accuracy and reliability of the results. In contrast to this study, another research project presented five classes for land suitability. These classes were labeled as very good, good, moderate, poor, and very poor.^[^ ^38^ ^]^ This classification system aims to provide a more detailed assessment of potential areas for RWH practices. Similarly, other studies have used yet another classification system. This system consists of five categories: unsuitable, poor, moderate, good, and excellent.^[^ ^39^ ^]^ This variety in classification systems underscores the complexity of assessing land suitability for rainwater harvesting and highlights the importance of using multiple methods for validation and classification in this field of research.

## 4 Conclusion

The research focuses on finding the best location for rural water harvesting (RWH) in the Konso zone of Ethiopia, known for its dry climate. The study utilizes spatial, field, and meteorological data to achieve this goal. An important aspect of the research involved correcting biases in the satellite‐based dataset known as CHIRP. After addressing these biases, the team evaluated the accuracy of CHIRP product in terms of rainfall data. Using a spherical variogram model, the study generated a detailed rainfall map. Indicating that the northern part of the study area is more suitable for RWH structures compared to other regions. Additionally, a LU/LC map was created, showing that shrubland is the most common land cover type in this area. The suitability map was developed by analyzing factors such as the rainfall map, proximity to agricultural land, and the topographic wetness index (TWI). Within this analysis, TWI was given the most significant weight at 23%. The research used a model builder in a GIS to effectively conduct the suitability analysis. The results revealed that only 0.1% of the study area is classified as very highly suitable, while 28.08% falls into the highly suitable category. A significant 63.16% of the area was deemed moderately suitable, and 2.63% was classified as low suitable.

The suitability map developed in this study has the potential to enhance agricultural productivity for farmers in the Konso zone. To validate the accuracy of the map, the research team compared it to the locations of existing RWH structures. The results were promising, showing that 92.68% of current structures are located within zones classified as highly or moderately suitable for RWH. In conclusion, the study demonstrates that combining MCA with GIS technology is an effective approach for identifying suitable sites for rural water harvesting in arid and semi‐arid regions lacking data. This research provides valuable insights for improving water management practices in similar climatic conditions.

## Conflict of Interest

There is no conflict of interest.

## Data Availability

The datasets used in the current study are available from the first and corresponding author on reasonable request.
